# The first dorsal metacarpal propeller perforator (FDMP) flap for finger reconstruction

**DOI:** 10.1080/23320885.2020.1806069

**Published:** 2020-08-14

**Authors:** Pietro G. di Summa, Kerry Davies, Andrew Hart

**Affiliations:** a Canniesburn Plastic Surgery Unit, Glasgow Royal Infirmary, Glasgow, UK; b Department of Plastic, Reconstructive and Hand Surgery, Centre Hospitalier Universitaire Vaudois (CHUV), Lausanne, Switzerland; c College of Medical Veterinary and Life Sciences, The University of Glasgow, Glasgow, UK

**Keywords:** First dorsal metacarpal artery, perforator flap, propeller

## Abstract

We describe here the first dorsal metacarpal artery propeller perforator flap, used to cover a full thickness, radiopalmar defect of the index finger after tumour excision. By associating a propeller design to the dissection of the first metacarpal pedicle, this flap can be effective in coverage of proximal index and web space defects, with primary closure and pleasant aesthetic outcomes. Harvested together with a superficial sensory branch from the radial nerve, this flap can provide effective coverage and sensory recovery.

## Introduction

Improved cutaneous vascular anatomical understanding, particularly the perforator paradigm, has expanded the armamentarium for upper extremity reconstruction [1]. Flaps should ideally match the tissues in the defect area, replacing like with like. This is especially true for hand and finger reconstruction, where thin, pliable, specialised skin is essential. Considering their better color and contour match, local flaps should be preferred [1].

Soft tissue defects in the hand can be covered by local transposition or rotation flaps, cross-finger flaps, or island flaps from adjacent rays. The greater laxity of dorsal skin enables dorsal hand flaps to provide moderate-sized single-stage reconstructions with primary donor site closure, and facilitates early mobilisation [2]. Among their advantages are the colour and texture match, preservation of axial pedicles if used as ‘propeller’ flaps, and broad anatomical applicability using the ‘freestyle’ concept [3].

Dorsal perforating branches emerge from the deep palmar arch supply skin over the distal hand and proximal phalanx [1]. The dorsal metacarpal arteries (DMCA) supply to the dorsal hand, and the distal DMCA perforator described by Quaba and Davison vascularises the distally based flap from the IInd-IVth intermetacarpal spaces [4].

Although dorsal metacarpal artery perforator propeller flaps have been described based on 2nd, 3rd and even 4th dorsal metacarpal (DMC) arteries, no reports to our knowledge focused on perforator flaps from the 1st DMC artery or first dorsal metacarpal artery (FDMA). Here we report a freestyle perforator propeller flap based on the FDMA that avoids dissection within the finger, and enables primary closure. A full thickness defect on the radial side of the IInd ray was reconstructed, and inclusion of a sensory branch of the radial nerve maintained sensory discrimination.

## Case report

**Figure 1.**
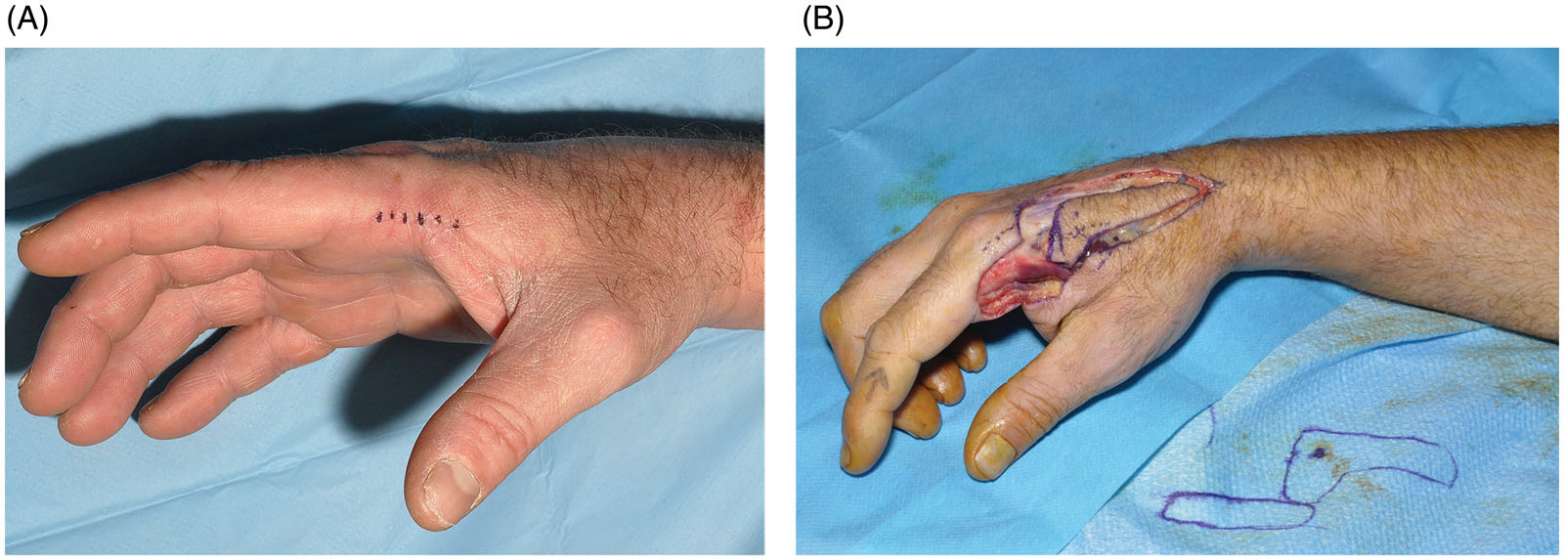
(A) Previous scar following diagnostic biopsy of atypical fibrous hystiocytoma, requiring wide local excision. (B) propeller flap design.

A 46 year old right-handed male manual worker with an incompletely excised atypical fibrous hystiocytoma of the palmo-radial aspect of his index finger/IInd MP joint proceeded to wide local excision. Resection included fascia overlying adductor pollicis and 1st dorsal interosseus, and adventitia surrounding the index finger’s radial neurovascular bundle (Figure 1(A)). A 2x4cm defect resulted, for which flap reconstruction offered the best cosmesis, hand function, and pain profile.

The FDMA perforator was localised by surface markings and a handheld doppler (Dopplex D900, Huntleigh Healthcare Ltd., U.K.). A single perforator propeller flap transfer was planned in reverse (Figure 1(B)). Surgery was performed under tourniquet control (250 mmHg) and loupe magnification (x3.5).

**Figure 2.**
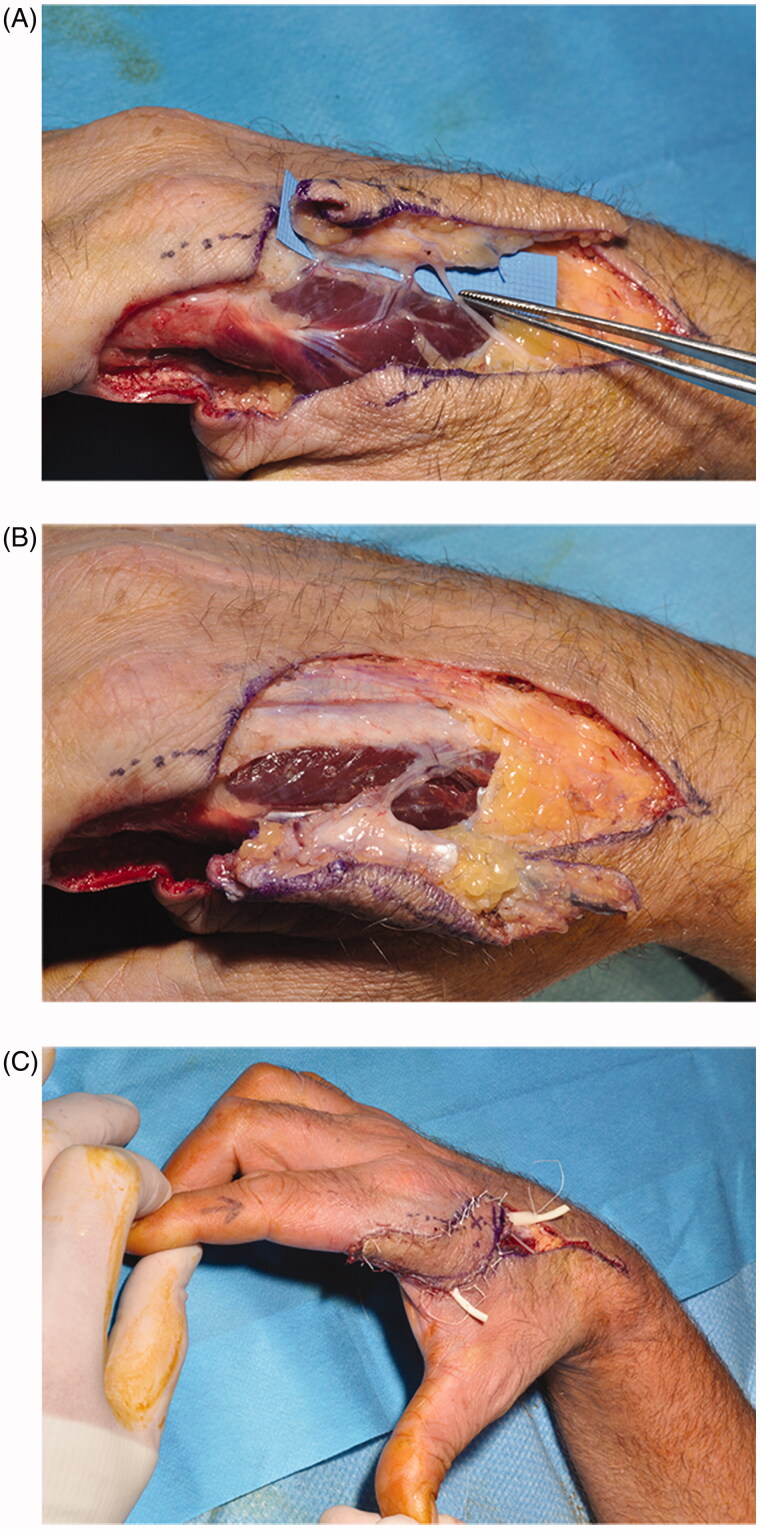
(A) FDMAp flap raised including sensory nerve branch. (B) proximal dissection of the FDMA to achieve greater reach and allow innervation to be preserved. (C) propeller rotation around the FDMA pedicle, and inset.

The dominant, distal perforator (metacarpal neck level) was exposed above the deep fascia. Proximal suprafascial dissection then defined a more proximal perforator and a small cutaneous nerve (Figure 2(A)). In order to incorporate these the design was refined (freestyle approach), the FDMA raised by subfascial dissection to the base of the first web space, and transposed palmarly to optimise reach (Figure 2(B)). The flap was rotated clockwise around the loosely curved FDMA to maintain innervation and both perforators avoiding pedicle kinking or compression. Insetting was performed (5/0 Vicryl Rapide, Ethicon) over small slips of Penrose drain, and the tourniquet deflated (Figure 2(C)). The donor site was closed primarily. A subcutaneous vein draining the tip of the flap was ligated and externalised in case of later venous congestion, but was not required. The hand was immobilised in a volar splint and elevated. After 24 h flap monitoring the patient was discharged. Mobilisation under hand therapy guidance started after one week.

**Figure 3.**
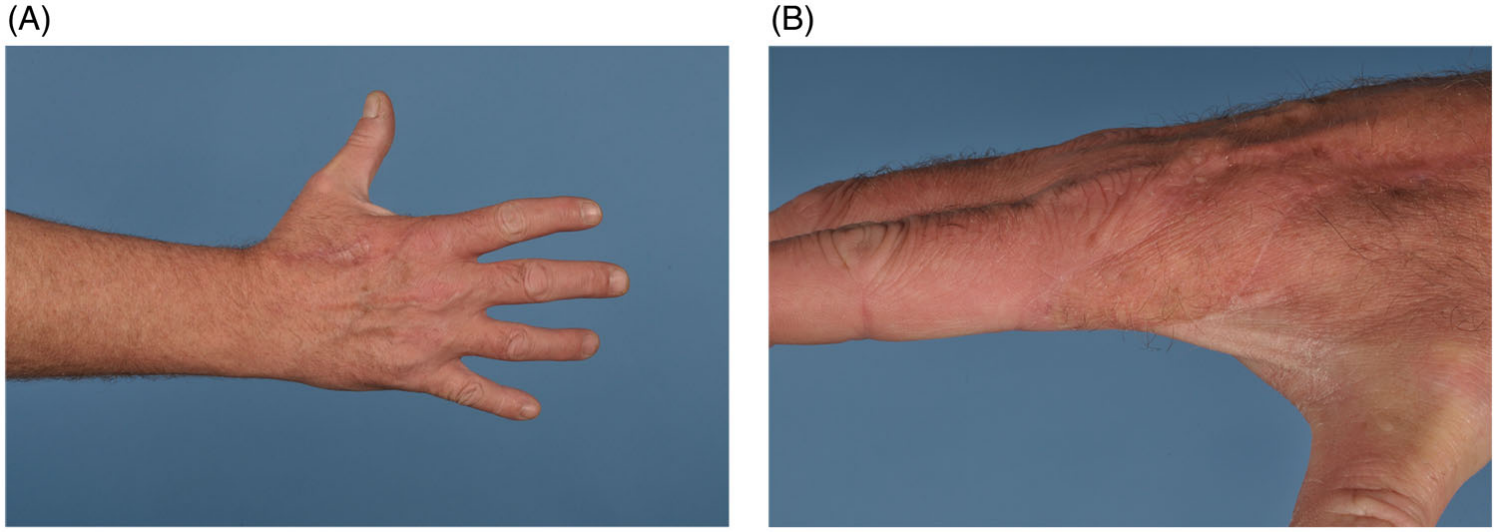
Postoperative results at 6 months, with full range of motion, and pleasant aesthetic outcome without donor site morbidity.

No complication arose, other than mild hypertrophic scar reaction predictable from the patient’s skin type, which settled with topical treatment. Full function was regained (Figure 3). The patient returned to heavy manual work. Static 2-point discrimination was <6 mm. Full range of movement was present in all joints (passive and active range goniometry). Jamar grip strength normalised (power grip: left = right: median 43 kg force; pinch grip: left = right: median 9 kg force).

## Discussion

Perforator-based flaps have been used in almost all body areas [5], according to the perforasome concept [6]. Raising the flap from the hand (FDMP flap) rather than from the finger reduces donor morbidity and enables primary closure, which supports early mobilisation to the overall benefit of the hand.

This report simply extends the literature on this paradigm to include the first webspace region of the hand. Although the present report does not rely on a cadaver study to define perforator locations and dimensions, this FDMA perforator propeller flap is conceptually comparable to DMCA perforator flaps based on metacarpals 2,3 and 4, with its dominant perforator arising at the metacarpal neck [7,8].

In DMCA perforator flaps, dorso-palmar anastomoses link the deep palmar system and superficial dorsal systems, enabling reverse flow flaps with more distal pivot points. ‘Reverse’ and ‘extended reverse’ versions of the DMCA flap are described, depending of the location of the nourishing perforator (proximal and distal to the metacarpal neck, respectively) [9,10].

Reverse flow flap elevation may be tedious since the DMCA lies deep to the extensor tendon, or even within the interosseus muscle [2] and it varies in diameter, the 1st and 2nd DMCA being more reliable [11]. In contrast, dorsal perforators arising proximal to the second metacarpal neck (Figure 1) allow simple dissection of propeller flaps that can reach the PIP joint level.

The FDMP presents the following advantages: completion of the surgical procedure under single brachial block and tourniquet control; thin skin matching the digit; vascular pedicle reliability; potential to preserve the FDMA; potential to remain sensate; index finger not transgressed; and primary closure of the donor site (assessable by pinch test, likely maximum width 3–4 cm) with minimal morbidity.

Raising the FDMA will enable greater reach, if required, or enable use of two perforators/sensory innervation. For a different defect (e.g. index finger proximal phalanx, narrower defect over radial aspect of IInd MP joint) a simple suprafascial propeller flap would have been employed based on the dominant perforator over the dorsoradial aspect of the IInd MC neck. This flap results more advantageous also when compared with a reversed dorsal metacarpal island flap as it is independent of the continuity of the FDMA and its communications with other arteries in distal site, which makes this flap a better choice for all defects of proximal index finger.

In our case, sensate reconstruction was felt beneficial in order to optimise keypinch, minimise the risk of neuroma pain, and best support use of handled tools. The sensory outcome matched that reported for standard and reverse flow FDMA flaps [12,13].

By sparing the index finger from dissection and avoiding donor site skin grafting, this flap should have less risk of inducing stiffness, cosmetic impairment, extensor tethering, or pain syndromes, and better facilitates aggressive early mobilization to the overall benefit of the hand.

## Conclusion

The described technique delivers this for proximal defects of the index finger, first webspace (and potentially of the first MCP joint at the thumb), and facilitates early mobilisation. Optimal aesthetics and functional recovery can be expected, particularly after primary closure of the low cost donor site.

Even if this flap would not reach defects distal to the proximal interphalangeal joint of the index (or radial aspect of the thumb), it offers microsurgeons a low-cost reconstructive option for a critical area of the hand, and for the supramicrosurgeon could represent a new regional donor site for a small free flap. For suitable IInd ray defects the flap could simply be propellered on a single perforator. Subcutaneous veins could be included for supercharging or free tissue transfer.
